# A Case of Chronic Intestinal Pseudo-Obstruction Managed With Percutaneous Endoscopic Transgastric Jejunostomy (PEG-J)

**DOI:** 10.7759/cureus.108142

**Published:** 2026-05-02

**Authors:** Koki Sato, Akinori Sekioka, Syusuke Yasuoka, Ryotaro Kabai, Kunihiko Tsuboi

**Affiliations:** 1 Gastroenterological Surgery, Osaka Saiseikai-Noe Hospital, Osaka, JPN

**Keywords:** chronic intestinal pseudo-obstruction (cipo), home parenteral nutrition, intestinal decompression, percutaneous endoscopic transgastric jejunostomy (peg-j), small bowel volvulus

## Abstract

Chronic intestinal pseudo-obstruction (CIPO) is a rare, debilitating gastrointestinal motility disorder characterized by symptoms of intestinal obstruction in the absence of a mechanical cause. Although no definitive treatment currently exists, multidisciplinary therapies such as percutaneous endoscopic transgastric jejunostomy (PEG-J) for intestinal decompression have improved symptom control, enabled the partial resumption of oral intake, and facilitated a transition to outpatient management. We report the case of a 65-year-old woman who was diagnosed with CIPO following emergency surgery for a small bowel volvulus. Given her progressive symptoms requiring sustained intestinal decompression, PEG-J was placed. Intermittent decompression via PEG-J resulted in considerable symptom relief, the partial resumption of oral intake, and a reduced need for repeated hospitalization. Owing to high-output drainage of approximately 2,000 mL/day, home parenteral nutrition was initiated in combination with PEG-J, enabling a successful transition to home-based care.

## Introduction

Chronic intestinal pseudo-obstruction (CIPO) is a rare gastrointestinal motility disorder characterized by symptoms suggestive of intestinal obstruction in the absence of mechanical obstruction [1]. This condition results from the dysfunction of the intestinal smooth muscle or the enteric nervous system. CIPO is classified as either primary or secondary; secondary CIPO occurs in association with other systemic, metabolic, or organic diseases, such as systemic sclerosis or amyloidosis [2,3]. Since no curative treatment exists for CIPO, management is primarily supportive and includes pharmacological therapies, nutritional support, and intestinal decompression. However, long-term use of a transnasal tube for intestinal decompression is often poorly tolerated and may hinder the transition to home-based care.

In recent years, percutaneous endoscopic transgastric jejunostomy (PEG-J) has been reported as a therapeutic option that allows intermittent intestinal decompression and may facilitate long-term outpatient management [4,5]. Compared with nasojejunal tubes, PEG-J offers better patient tolerance and allows for more controlled drainage. In addition, it may be preferable to surgical options such as decompressive ileostomy, as it is less invasive and better suited for long-term management, particularly for controlling the amount of drainage. We report a case of CIPO that required surgical intervention for a small bowel volvulus, in which intermittent intestinal decompression using PEG-J contributed to symptom relief, avoided multiple surgeries, and facilitated the transition to home care.

## Case presentation

A 65-year-old woman with lower abdominal pain, distension, and nausea was referred to our hospital’s emergency department. She had a history of well-controlled hypothyroidism and a prior episode of intestinal dilation with pneumoperitoneum, which had been treated conservatively one year prior. Following this episode of intestinal dilation, the patient developed chronic abdominal bloating and constipation. Her height and weight were 158 cm and 41 kg, respectively. Her blood pressure was 110/64 mmHg, pulse rate was 82 beats/min with a regular rhythm, and body temperature was 36.7 °C.

**Figure 1:**
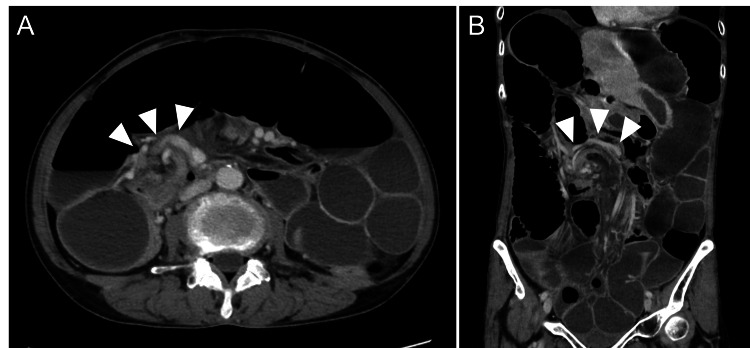
CT showing small bowel volvulus (A) Axial CT image showing the whirlpool sign of the mesentery (arrowheads), indicating mesenteric torsion. (B) Coronal CT image showing the same finding (arrowheads) CT: computed tomography

The abdomen was mildly distended with tenderness, primarily in the lower abdomen, and bowel sounds were decreased. Laboratory examinations showed mildly elevated inflammatory markers that were not suggestive of infection or ischemia: white blood cell count of 6,300/μL (normal range: 3,500-8,000/μL) and C-reactive protein level of 0.4 mg/dL (normal range: <0.3 mg/dL). CT revealed marked dilatation of the small intestine with abnormal bowel configuration, suggesting a strangulated small bowel obstruction secondary to small bowel volvulus (Figure 1). Based on these findings, a diagnosis of strangulated small bowel obstruction was made, and emergency laparotomy was performed.

Intraoperative findings showed marked dilatation and thinning of the small intestinal wall, approximately 100 cm proximal to the terminal ileum. A small bowel volvulus of approximately 720° was identified around the root of the mesentery, with the pivot point located approximately 170 cm proximal to the terminal ileum. Since no obvious ischemic changes were observed in the involved bowel, detorsion was performed without intestinal resection. Table 1 presents the laboratory findings.

**Table 1:** Laboratory data WBC: white blood cell; RBC: red blood cell; HGB: hemoglobin; HCT: hematocrit; CRP: C-reactive protein; AST: aspartate aminotransferase; ALT: alanine aminotransferase; T-BiL: total bilirubin; D-BiL: direct bilirubin; BUN: blood urea nitrogen; Na: sodium; Ki: potassium; Cl: chloride; Lac: lactate

Test (unit)	Admission value	Reference range
WBC (x10^3^/µL)	6.3	3.5-8.0
RBC (x10^6^/µL)	4.82	3.8-4.8
HGB (g/dL)	14.6	11.3-14.9
HCT (%)	44	36.0-47.0
Platelets (x10^3^/µL)	279	120-400
CRP (mg/dL)	0.4	<0.3
Total protein (g/dL)	7.9	6.5-8.2
Serum albumin (g/dL)	4.9	3.7-5.5
LDH (U/L)	195	124-222
AST (IU/L)	32	10-40
ALT (IU/L)	29	5-45
T-BiL (mg/dl)	0.6	0.4-15
D-BiL (mg/dl)	0.2	0.0-1.4
BUN (mg/dl)	24.5	8.0-20.0
Serum creatinine (mg/dL)	1.07	0.5-0.8
Na (mEq/L)	142	136-148
K (mEq/L)	4.6	3.6-5.0
CI (mEq/L)	102	97-108
Lac (mmol/L)	2.1	0.5-1.6

**Figure 2:**
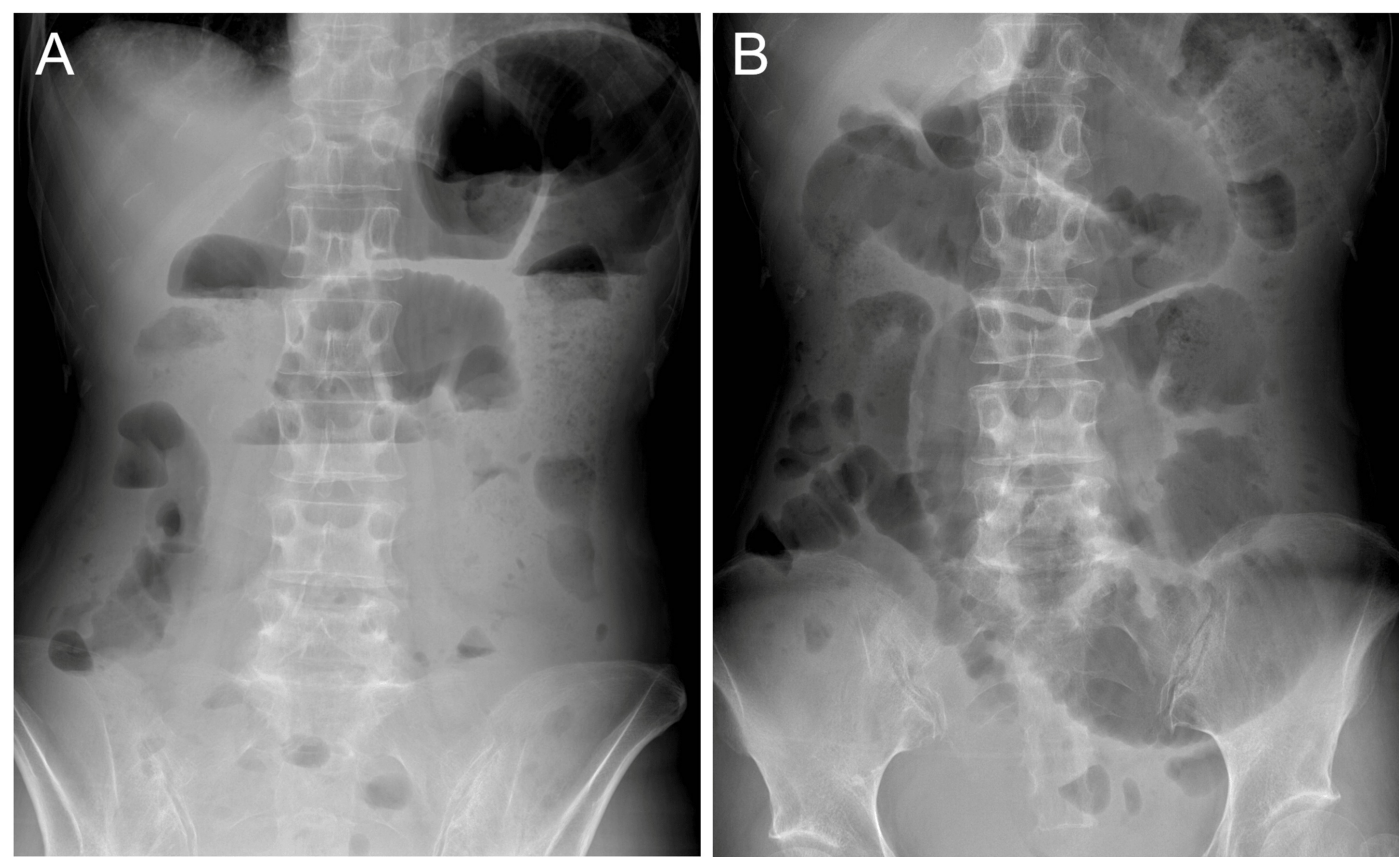
Abdominal radiography showing small bowel dilation with multiple air-fluid levels (A) Abdominal radiograph in the standing position demonstrating air-fluid levels. (B) Abdominal radiograph in the supine position, highlighting bowel dilatation

Postoperatively, intestinal dysmotility persisted, and the resumption of oral intake was delayed. During this admission, Raynaud’s phenomenon and markedly elevated anti-centromere antibody levels (1,280 U/mL) were detected; however, the diagnostic criteria for systemic sclerosis were not fulfilled because skin sclerosis was absent. With conservative management, the oral intake gradually improved, and the patient was discharged on postoperative day 30. One month after discharge, outpatient abdominal radiography showed persistent marked small bowel dilatation with multiple air fluid levels (Figure 2).

**Figure 3:**
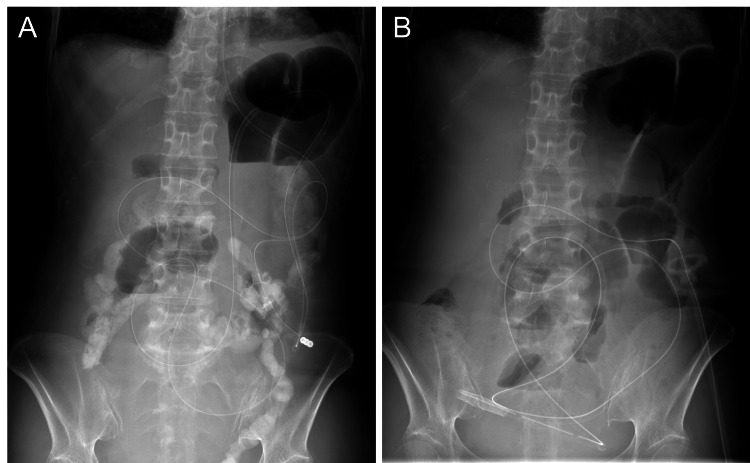
Abdominal radiography after nasojejunal tube placement and PEG-J (A) Abdominal radiograph (standing position) after nasojejunal tube placement showing partial reduction of air-fluid levels. (B) Abdominal radiograph (standing position) after replacement with PEG-J PEG-J: percutaneous endoscopic transgastric jejunostomy

**Figure 4:**
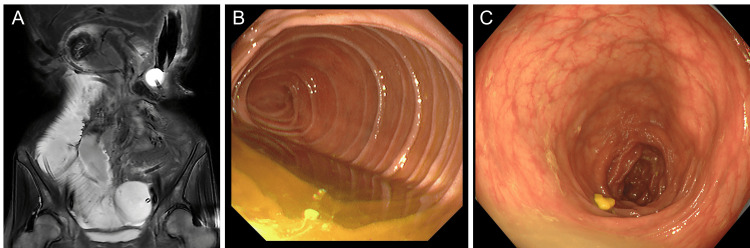
Various examinations to assess the digestive tract (A) Cine MRI showing markedly decreased peristaltic activity with absent propagating contractions. (B) Esophagogastroduodenoscopy showing no mechanical obstruction. (C) Colonoscopy showing no mechanical obstruction MRI: magnetic resonance imaging

Two months after discharge, abdominal pain and vomiting recurred, and the patient was readmitted. On readmission, the abdomen was distended without Blumberg’s sign. Abdominal CT revealed small bowel dilatation without evidence of definite mechanical obstruction or strangulation. Therefore, decompression was initiated using a nasojejunal tube (Figure 3A). Although abdominal distension and nausea improved after decompression, small bowel dilatation persisted on radiography. Cine MRI revealed markedly decreased peristaltic movement throughout the small intestine (Figure 4A). Esophagogastroduodenoscopy, colonoscopy, and small bowel follow-up did not reveal any mechanical obstruction (Figures 4B, 4C). Based on these findings, a diagnosis of CIPO was made [6].

Since long-term intestinal decompression was deemed necessary, a PEG-J tube (GB Jejunal Tube®, 120 cm, Fuji Systems, Tokyo, Japan) was placed instead of the nasojejunal tube. After PEG-J placement, the patient tolerated small amounts of oral intake, and bowel movements and passage of flatus gradually improved with intermittent manual decompression (Figure 3B). However, oral intake alone did not achieve stable hydration and nutritional intake, mainly due to the high daily PEG-J output of approximately 2,000 mL, which was necessary for symptom relief. Therefore, a central venous port (Orphis CV Kit Neo®, SB KAWASUMI, Kanagawa, Japan) was placed, and home parenteral nutrition was initiated. This multidisciplinary approach enabled discharge to home care.

## Discussion

CIPO is a rare disorder characterized by symptoms of intestinal obstruction caused by impaired gastrointestinal motility in the absence of mechanical obstruction [1]. This condition is classified as primary or secondary CIPO, with secondary forms being associated with a range of underlying disorders such as collagen vascular diseases, particularly systemic sclerosis, as well as hypothyroidism, diabetes mellitus, and mitochondrial disease [7,8]. Recently, proposed diagnostic criteria for CIPO have included symptoms of intestinal obstruction (e.g., abdominal pain and bloating), bowel dilatation or air-fluid levels detected by various methods, and the absence of structural abnormalities explaining the dilated loops [6]. 

The management of CIPO primarily consists of supportive therapies, including intestinal decompression, nutritional support, and pharmacological treatments. No definitive curative treatment has been established, and surgical interventions such as resection of dilated bowel segments are considered beneficial only in selected cases [8]. Therefore, the treatment must be tailored to each patient based on disease severity, progression, and associated underlying conditions.

Our patient required surgical intervention due to small bowel volvulus during the clinical course. At that time, the volvulus was considered the cause of intestinal dilation; however, persistent postoperative intestinal dilatation and recurrent symptoms suggested an underlying motility disorder that could not be explained by mechanical obstruction. Therefore, it is possible that the volvulus was preceded by bowel dilatation related to CIPO, although this interpretation remains speculative. Importantly, no definitive evidence of intestinal ischemia was observed on imaging or intraoperative findings. In addition, mildly elevated laboratory values such as BUN and lactate were considered to reflect intravascular dehydration rather than findings suggestive of intestinal ischemia. Based on these findings, the patient was diagnosed with CIPO after excluding mechanical obstruction and ischemia.

Long-term intestinal decompression can become necessary at some stages of CIPO management to relieve symptoms and maintain quality of life; however, the access route of decompression is still debatable. Conventional nasojejunal tubes are often poorly tolerated during long-term use and may limit the patients’ quality of life and the feasibility of home care. Decompression ileostomy is a palliative option for patients who are refractory to treatment [9]. However, an ileostomy cannot control the drainage volume, potentially causing dehydration and electrolyte imbalance.

PEG-J enables more controllable intestinal decompression without the need for a transnasal route and enables easier adjustment of the drainage volume. This approach may be useful for long-term management and the transition to home care; however, reports on PEG-J decompression in patients with CIPO are limited [4,5,10]. In our case, intermittent intestinal decompression using PEG-J relieved abdominal symptoms, avoided repeat laparotomies, and enabled the resumption of oral intake. Previous studies have highlighted certain drawbacks of PEG-J placement, such as dehydration or electrolyte imbalance due to excessive drainage and peristomal skin complications [4]. In this case, the amount of drainage through the PEG-J required careful adjustment, and approximately 2,000 mL of daily drainage was necessary to control the abdominal symptoms. Therefore, careful fluid and electrolyte management was required. Additionally, the long-term outcomes of PEG-J placement in patients with CIPO remain uncertain due to the rarity of the condition, and further research is needed to better evaluate its effectiveness.

## Conclusions

This report describes a rare case involving the use of PEG-J decompression in a patient with CIPO. In our patient, a multidisciplinary approach, including PEG-J and home parenteral nutrition, contributed to effective disease control and improved quality of life. Intestinal decompression using PEG-J in patients with CIPO and progressive abdominal symptoms may serve as a valuable therapeutic option for symptom relief and facilitation of transition to home care.
